# The oxytocin receptor signalling system and breast cancer: a critical review

**DOI:** 10.1038/s41388-020-01415-8

**Published:** 2020-08-11

**Authors:** Huiping Liu, Christian W. Gruber, Paul F. Alewood, Andreas Möller, Markus Muttenthaler

**Affiliations:** 1 Institute for Molecular Bioscience, The University of Queensland, Brisbane, QLD 4072 Australia; 2 Center for Physiology and Pharmacology, Medical University of Vienna, 1090 Vienna, Austria; 3 Tumour Microenvironment Laboratory, QIMR Berghofer Medical Research Institute, Herston, QLD 4006 Australia; 4 Institute of Biological Chemistry, Faculty of Chemistry, University of Vienna, 1090 Vienna, Austria

**Keywords:** Breast cancer, Hormone receptors, Mechanisms of disease, Target identification

## Abstract

Breast cancer is making up one-quarter of all new female cancer cases diagnosed worldwide. Breast cancer surgeries, radiation therapies, cytotoxic chemotherapies and targeted therapies have made significant progress and play a dominant role in breast cancer patient management. However, many challenges remain, including resistance to systemic therapies, tumour recurrence and metastasis. The cyclic neuropeptide oxytocin (OT) elicits a plethora of biological responses via the oxytocin receptor (OTR) in both the central and peripheral nervous system, including social bonding, stress, maternal behaviour, sexual activity, uterus contraction, milk ejection and cancer. As a typical member of the G protein-coupled receptor family, OTR represents also an intriguing target for cancer therapy. There is emerging evidence that OTR plays a role in breast cancer development and progression, and several breast cancer cell lines express OTR. However, despite supporting evidence that OT lowers breast cancer risks, its mechanistic role in breast cancer development and the related signalling pathways are not fully understood. Here, we review the current knowledge of the OT/OTR signalling system in healthy breast tissue as well as in breast cancer, and discuss OTR as a potential therapeutic target for breast cancer management.

## Introduction

Breast cancer is the most commonly diagnosed cancer in women, making up one-quarter of all new female cancer cases diagnosed worldwide [1]. Current treatments for breast cancer include surgery, systematic radio- and chemotherapy, hormonal therapy, targeted therapy (such as HER2- and EGFR-targeted therapy) and immunotherapy [2]. Progress in clinical management strategies and early detection through increased awareness and use of mammography has improved survival for female breast cancer patients, with 5-year relative survival rates of 89% [2]. However, for metastatic breast cancer, the 5-year relative survival rate remains low at 27% [3].

The oxytocin receptor (OTR) is a G protein-coupled receptor (GPCR) of the class A/rhodopsin family and a key receptor system for birth, breastfeeding and social interactions, particularly for mother–child bonding [4, 5]. It is activated by its endogenous ligand oxytocin (OT), a cyclic nonapeptide (CYIQNCPLG) with a disulfide bond between the two cysteine residues and a C-terminal amide. The OT/OTR signalling system is an evolutionary ancient (600–700 million years [6]) and widely distributed signalling system that regulates a multitude of functions in a wide range of different cell types [5, 7, 8]. It is utilised by many cancer types [9–11], including small-cell lung carcinoma [12], trophoblast and choriocarcinoma [13], osteosarcoma [14], Kaposi’s sarcoma [15], neuroblastoma and glioblastoma [16], endometrium adenocarcinoma [17], leiomyoma [18], ovarian carcinoma [19], prostate carcinoma [20, 21] and osteosarcoma [22]. The connection between breast cancer and the OT/OTR signalling system emerged from epidemiologic studies indicating that childbearing and breastfeeding reduce the risk of breast cancer development [23, 24]. Dysregulation of OT/OTR in breast tumour tissues could also be linked with the immune escape mechanism of the cancer cells considering its functions in the immune system [25, 26]. A range of in vitro and in vivo studies support this OT/OTR involvement in breast cancer, which is the focus of this review. We furthermore review OTR’s signalling pathways to clarify its regulatory role in cell growth and OTR’s therapeutic potential for breast cancer management.

## OT in human breast development and tumorigenesis

The mammary gland is one of the few organs that undergoes most of its development postnatally through distinct stages (embryonic, pubertal growth, pregnancy, lactation, and involution stages) and breast development continues throughout life [27]. Mammary cells undergo tightly regulated changes in growth, differentiation, invasion, and apoptosis, and dysregulation of these processes can be a contributor to breast tumorigenesis [28]. Breast cancer risk is affected by a variety of factors, including reproductive history and lifetime hormonal exposure, which also influences the course of the disease [29]. Oestrogen, progesterone and prolactin are well-studied hormonal regulators of mammary gland development [27, 29, 30]; less is known however about the regulatory roles of OT/OTR during breast development and tumorigenesis.

### OT and breast development

The effects of OT on non-lactating mammary gland remains poorly explored since most studies have focused on OT’s role during pregnancy and lactation [31]. Breast development culminates during pregnancy and the lactation cycle when the mammary gland undergoes complete remodelling, including maturation into a functional milk-secretory organ. This is followed by regression as weaning commences, which is completed after involution during which the breast regresses to a resting state [32]. Normally, mammary glands of lactating rats enter involution following the cessation of suckling, however, OT administration in lactating rats after removal of their litters (which mimics the weaning process) results in a delay of involution [33], an effect also observed in other studies [34–36]. These data suggest a role of OT in maintaining and modulating the status of the mammary gland.

OT is also produced, and secreted by myoepithelial and epithelial cells within normal and neoplastic breast [37], supporting the existence of local autocrine/paracrine loops that could influence cell growth, and differentiation of the mammary gland during breast development. In fact, OT can affect breast cell growth and differentiation both in vitro and in vivo [38–40]. For example, exposure of myoepithelial cells to OT results in the enhancement of myoepithelial cell differentiation and proliferation, enhancing the effect of mammotrophic hormones in non-lactating mouse mammary gland, with a less pronounced effect in luminal breast epithelial cells [38]. OT is also required for postpartum mammary proliferation, supported by the observation that mammary epithelial cells in OT-deficient mice fail to proliferate within 12 h after parturition, while ~2% of the alveolar cells in wild-type animals incorporated DNA and proliferated. Furthermore, the continuous suckling of pups leads to lobulo-alveolar expansion in control animals but not in OT-deficient animals [39].

### Protective effects of OT against breast cancer development

OT’s preventive potential against breast cancer was initially discussed and reviewed in the 1990s, based on the hypothesis that OT production from nipple stimulation leads to myoepithelial cell contractions that help to relieve acinar gland distension and to remove carcinogenic substances such as superoxide free radicals from the breast, thereby reducing the risk to develop breast cancer [41].

Childbearing and breastfeeding, where OT plays a crucial role, are known protective factors against breast cancer [23, 24]. Childless women (the nulliparous populations) have a higher risk for breast cancer development than those who have given birth to one or more children [41–43]. Prolonged breastfeeding, usually involving multiple children, correlates with a progressive decrease in breast cancer risk [23, 44, 45]. In the collaborative reanalysis of individual data from 47 epidemiological studies in 30 countries, including 50,302 women with and 96,973 women without breast cancer, the relative risk to develop the disease decreased by 4% for every 12 months of breastfeeding, and by 7% for each birth in women who never breastfed [24]. In a study from Sri Lanka, which included 100 cases of breast cancer and 203 controls, the risk was reduced even more profoundly, with 12–23 months, 24–35 months and 36–47 months of breastfeeding reducing breast cancer risk by 66%, 87% and 94%, respectively (compared to 0–11 months of breastfeeding) [46]. The ‘non-sexual’ breast may carry a breast cancer risk similar to that of the ‘non-breastfeeding’ breast [47]. In age-matched nulliparous women, breast cancer incidence was lower in the sexually active group than in the celibate group [43], which also aligns with increased OT plasma levels in sexually aroused women [48].

Physical exercise is another protective factor [49–51] that correlates with increased OT levels [52, 53] with an average risk reduction is 25% among physically active women compared to the least active women [54]. Physical exercise also reduces symptoms in cancer patients (including breast cancer patients) and improves their physical and psychosocial state during and after treatments [55]. A mouse xenograft model study demonstrated that OT can mediate the beneficial effects of physical exercise on breast cancer growth. Both exercise and OT administration decreased tumour volume and weight, and OT plasma concentration in the tumour and exercise group was more than 10-fold higher than in the tumour control group (tumour without exercise) [56]. OT levels in the pituitary and blood also increased in a rat breast cancer model induced by N-methyl-nitrosourea (NMU)-treatment due to inhibition of insulin-regulated aminopeptidase (an oxytocinase) and decrease of catabolism of OT in the hypothalamus [57]. This is consistent with a human study, which observed higher OT plasma levels in breast cancer patients (*n* = 40) than in healthy individuals [58]. Higher OT plasma levels, however, do not mean higher OT levels in breast cancer tissue. OT levels in breast tumour tissue is ~2-fold lower than in normal breast tissue [59], which may be related to increased activity of the oxytocinase in breast cancer tissues [60]. A reduction of OT in the mammary tissue may partially account for mammary tumorigenesis, while the increases in the pituitary release of OT may serve as a compensatory mechanism to suppress the proliferation of breast cancer [26]. Thus, an increase in OT levels might serve as a potential biomarker for breast cancer development or progression.

The exact mechanisms of these protective effects and the interplay with other factors in these physiological situations remain to be fully elucidated. However, OT is associated with these effects due to its increasing levels in plasma during the first to third trimester of pregnancy [61], breastfeeding [62], sexual response [48] and physical exercise [52, 53]. OT is also a potent anti-inflammatory agent that can attenuate oxidative stress and inflammatory cytokine release in colonic injury [63], inflammation of the renal parenchyma [64], and vascular diseases [65–67]. Since oxidative stress and inflammation are two crucial contributors to many diseases, including breast cancer [26], the anti-inflammatory effects of OT should be taken into consideration for its potential to reduce the risk for breast cancer development and disease progression. While the preventive effects of OT look promising, its exact role in reducing breast cancer risks requires further investigation.

## OTR expression and function in the breast

OTR’s function of mediating the effects of OT in the normal female breast is well understood. OT and OTR not only regulate lactation but also control the organogenetic process and the histogenesis of different lesions in the breast [68], for instance, OTR overexpression can induce abnormal mammary gland development [69]. There is emerging evidence that OTR also plays a role in breast cancer development, however, how receptor expression and signal transduction change when breast cancer initiates remains unclear.

### OTR in the healthy breast

**Fig. 1:**
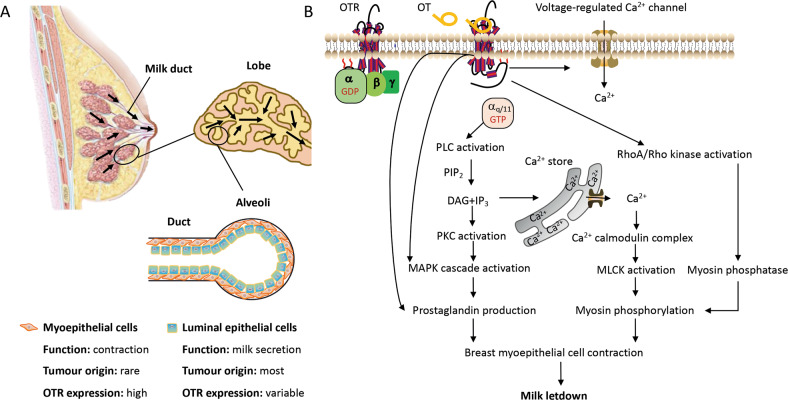
Oxytocin receptor in the female breast and its function in mediating milk letdown. **a** Basic components of a mature female mammary gland, with black arrows indicating milk flow. **b**. Signal transduction mechanism of OTR during lactation. OTR coupling to Gα_q/11_ under the stimulation of OT activates phospholipase C (PLC), which hydrolyses phosphatidylinositol biphosphate (PIP_2_) to diacylglycerol (DAG) and inositol triphosphate (IP_3_). DAG activates protein kinase C (PKC), while IP_3_ causes release of Ca^2+^ from the sarcoplasmic reticulum Ca^2+^ store. Gα_q/11_ also causes activation of voltage- and agonist-regulated Ca^2+^ channels, which allow Ca^2+^ influx into the cells. Intracellular Ca^2+^ binds to calmodulin forming the Ca^2+^/calmodulin complex, which activates myosin light-chain kinase (MLCK), resulting in myoepithelial cell contraction. OTR and PKC can activate the mitogen-activated protein kinase (MAPK) cascade, resulting in prostaglandin production, which also contributes to the contractile effect. Consequently, the alveolar milk is shifted to the cisternal space, drained into the lobule lumen towards the nipple through the lactiferous duct. The anatomy of the female breast presented in Fig. 1a was adapted from [71].

OTR is endogenously expressed in most cells at low levels (often <100 fmol/mg protein) [70], but it is most abundant in the female breast [10]. In normal non-lactating and non-neoplastic human breast specimens, OTR is located on the cell membrane and in the cytoplasm of cells of the basal cell layer, which mainly consists of myoepithelial cells and undifferentiated cells, with very few epithelial cells being OTR positive (Fig. 1a) [68]. The basic components of a mature mammary gland are the alveoli lined with milk-secreting cuboidal cells and surrounded by myoepithelial cells. These alveoli form groups known as lobules. Each lobule has a lactiferous duct that connects to the nipple (Fig. 1a) [71]. The primary function of OTR in the breast is to mediate milk ejection during breastfeeding. This has been confirmed in both OTR-deficient [72] and OT-deficient [73] mice, which fail to nurse their offspring due to impaired milk ejection and maternal nurturing. OTR expression remains up-regulated from pregnancy to lactation in mammary gland in rats [74]. Both mammary gland OTR expression and OT plasma level rise in response to breastfeeding, an effect that persists during the lactation period [62, 75]. The contractility response of the myoepithelial cells is mainly mediated by OTR coupling to Gα_q/11_ under the stimulation of OT (Fig. 1b) [76, 77], which increases the intracellular calcium ([Ca^2+^]_i_) concentration by releasing Ca^2+^ from internal stores, as well as by mobilising extracellular pools via the opening of voltage- and agonist-sensitive Ca^2+^ channels [78, 79]. Consequently, the alveolar milk is shifted to the cisternal space, drained into the lobule lumen towards the nipple through the lactiferous duct [80]. OTR and protein kinase C (PKC) activation can activate the MAPK cascade resulting in prostaglandin production, which contributes to contraction and milk ejection [81, 82]. Moreover, OTR can also activate the RhoA/Rho-kinase cascade resulting in increased myosin light-chain kinase (MLCK) phosphorylation responsible for OT-induced cell contractility [83]. Apart from Gα_q/11_ signalling, part of the OTR response in the uterus is also linked to OTR coupling to Gα_i_ [84], which is, however, less understood. Meanwhile, co-stimulation of the ACII-cAMP-PKA signalling cascade via Gα_i_-derived βγ-dimers can counterbalance the Gα_q/11_-dependent effect and self-limit the OT-induced cell contractile response [85]. In the central nervous system, Gα_q/11_ and βγ subunits play a dominant role in burst firing evoked by applied OT or by suckling in the milk-ejection reflex [86].

### OTR in breast cancer

OTR expression and function in breast cancer is unexpected because human breast cancers are typically of epithelial, not myoepithelial origin (Fig. 1a) [87]. OTR is widely present in breast cancer cell lines, benign sclerosing adenosis and breast carcinomas [68, 88–90]. So far there are more than ten human breast cell lines known to have positive OTR expression either at the protein or mRNA level, including MCF-7, T47D, Hs578T, SK-BR-3, MDA-MB4353, BT474, BT549, BT20, ZR75, MDA-MB-231, MDA-MB-361, MDA-MB-468, as well as non-cancer stromal cell lines, including HMEC (human dermal microvascular endothelial cells), and B-TEC (tumour-associated endothelial cells purified from human breast carcinomas) [37, 40, 59, 88, 91–94]. However, not all breast cancer tissues have detectable OTR expression, either at the mRNA level (positive in 20 out of 27, 74% [68]) or protein level (positive in 7 out of 13, 54% [68], 52 out of 57, 91% [88], and 15 out of 19, 79% [90]). So far there exists only one study indicating that OTR expression in tumour tissues is lower (>11-fold at mRNA level and >2-fold at the protein level, *n* = 4) than in normal contralateral breast samples from the same individual. The authors of this study stated that a reduction of OTR expression in breast cancer could be effective in promoting cancer progression [58].

No conclusive data exist yet for the diagnostic or prognostic value of OTR for breast cancer development or progression. In a canine mammary tumour study, OTR levels were higher in benign tumour tissues (6.42 ± 0.27, *n* = 19) than in malignant tumour tissues (5.75 ± 0.26, *n* = 24, *P* = 0.08), higher in malignant oestrogen receptor α (ERα)-positive tumour tissues (6.27 ± 0.21, *n* = 14) than in ERα-negative tumour tissues (5.54 ± 0.37, *n* = 10, *P* = 0.06), and significantly higher in histological grade I (6.2 ± 0.29, *n* = 10) and II (6 ± 0.53, *n* = 7) lesions than in grade III (4 ± 0, *n* = 3, *P* < 0.05) lesions [95]. In humans, very few studies have indicated a relationship between OTR expression and other clinical variables (such as tumour size, biomarkers, metastasis or histological grade), except that OTR is more likely to be correlated with oestrogen receptor-positive (ER^+^) breast tumours. For example, there were more samples diffusely expressing OTR (diffusely expressing was defined as that >80% cells were stained by IHC detection) in ER^+^ samples (18 out of 32 ER^+^ patients) than in ER-negative (ER^−^) patients (7 out of 19 ER^−^ patients) [88], with OTR gene expression being 8.6-fold higher in breast tumour tissues of ER^+^ patients (*n* = 27) than ER^−^ patients (*n* = 10) [58].

There is little knowledge about the regulation of the OT/OTR system during breast cancer development. Given the evidence gathered to date and the discussion above, understanding the OT/OTR system in breast cancer is of major biological and clinical significance.

## Preclinical studies of OTR ligands in breast cancer

The protective effects of OT along with OTR presence in breast cancer cell lines and tissues led to the investigation of OT and OT analogues (atosiban, vasopressin, desmopressin) in several breast cancer models. The study details and therapeutic implications for breast cancer management are summarised in the following paragraphs.

### In vivo studies

Targeting OTR for breast tumour growth inhibition and tumour reduction was studied in vivo in mouse mammary carcinoma TS/A [96], rat carcinoma D-R3230AC [96] and mouse MC4-L2 carcinoma [56, 97] (Table 1). In all of these studies, OT had profound effects on tumour growth inhibition [56, 68, 96, 97] or even tumour reduction [97], and OT was a key mediator for the observed beneficial effects of physical activity in breast cancer [56]. Of note, in the mouse MC4-L2 carcinoma xenograft model [56, 97], doses of 1,000-fold difference (from 0.03 to 30 μg/kg) were used. Although there are no specific guidelines with regards to the dose and dosing interval for long-term use of OT in cancer research, the published doses of the peptide need to be critically considered for any further studies defining tolerable and therapeutic doses.

**Table 1:** Overview of in vivo studies using OT analogues for breast cancer inhibition.

Species	Cell lines	Treatment	Tumour reduction	Reference
Mouse (BALB/c)	TS/A	OT pellet, steady 10^−8^ M^a^ (~0.58 μg/kg), 21 days	50%^b^	[68, 96]
		AT o.p., steady 10^−9^ M (~0.058 μg/kg) and 10^−8^ M^a^ (~0.58 μg/kg), 14 days	65%, 72%	[96]
Rat (Fisher)	D-R3230AC	OT o.p., steady 10^−8^ M^a^ (~0.64 μg/kg), 14 days	74%^c^	[96]
		AT o.p., steady 10^−9^ M (~0.058 μg/kg) and 10^−8^ M^a^ (~0.58 μg/kg), 14 days	66%, 90%^c^	
Mouse (BALB/c)	MC4-L2	OT, 0.03 μg/kg, daily i.p., 14 days	82%^b^	[56]
		AT, 1.5 μg/kg, daily i.p., 14 days	No effect	
Mouse (BALB/c)	MC4-L2	OT, 30 nmol/kg (~30 μg/kg), daily i.p., 14 days	49% volume^b^	[97]
		AT, 1.5 μg/kg, daily i.p., 14 days	No effect	
Athymic nude mice	MDA-MB-231	[V^4^Q^5^]-desmopressin, 0.3 μg/kg i.v. thrice weekly, in combination or not with weekly cycles of paclitaxel (10 mg/kg i.p.)	Inhibition of tumour growth and invasion, and enhanced chemotherapy effects	[152]^d^
Mouse (BALB/c)	F3II	[V^4^Q^5^] Desmopressin, 0.3 μg/kg i.v. thrice weekly, in combination or not with weekly cycles of carmustine (20 mg/kg i.p.)		

*OT* oxytocin, molecular weight = 1007.2 g/mol; *AT* atosiban, molecular weight = 994.2 g/mol; mouse blood volume ~58 mL/kg, rat blood volume ~ 64 mL/kg; *o.p.* osmotic pump, *i.p.* intraperitoneal injection, *i.v.* intravenous injection, alkylating drug carmustine and antimitotic agent paclitaxel are clinical drugs for cancer cytotoxic therapy.

^a^Steady plasma concentration obtained by treatment (pellet or osmotic pump).

^b^No exact number shown, calculated according to data or figure of publication.

^c^Percentage of tumour volume increase slower than controls, calculated from tumour volume increase compared with treatment day 1, tumour volume increase was 200% in controls, 52% in OT treated, 20% and 67% in AT 10^–9^ M and 10^–8^ M treated animals, all other inhibitory responses were compared with control group.

^d^Main receptor investigated was V_2_R, not OTR.

The OT analogue atosiban (AT) also reduced breast cancer tumour growth in the TS/A (mouse) and D-R3230AC (rat) xenograft models [96]. AT used to be considered an OTR antagonist, but was later characterised as a biased OTR ligand that blocks the Gα_q/11_ pathway and activates the Gα_i3_ pathway [98, 99]. By contrast, AT had no effects on tumour reduction in the mouse MC4-L2 carcinoma models, where OT reduced tumour growth [56, 97].

^111^In-labelled OT-like tracer ^111^In-DOTA-LVT (^111^In-1,4,7,10-tetraazacyclododecane-N,N’,N”,N”’-tetraacetic acid-Lys^8^-vasotocin) [100], and its improved version ^111^In-DOTA-dLVT (^111^In-1,4,7,10-tetraazacyclododecane-N,N’,N”,N”’-tetraacetic acid-Lys^8^-deamino-vasotocin) [101] were developed to visualise OTR-expressing breast tumours and to study tracer/ligand uptake in mouse xenograft models. The tumour/blood, tumour/liver and tumour/kidney uptake ratios of ^111^In-DOTA-dLVT were 7.58, 1.42, and 0.06, respectively, in the preclinical study of animals bearing TS/A tumours [101]. The reason for high ligand uptake in the kidney and liver is that OT metabolism occurs mainly in these organs [102]. These studies are important as they support the therapeutic potential of OTR in breast cancer management, in terms of reduction and imaging/diagnosis of OTR-positive breast tumours.

While these findings are promising, it should be noted that none of the in vivo studies used xenograft models with human breast cancer cell lines. To improve the translational perspectives, in vivo studies targeting OTR in human breast cancer xenograft models are required. OT, while important as the endogenous ligand, might also not be the ideal ligand for therapeutic development, considering that it also activates the three to OTR closely related vasopressin receptors (V_1a_R, V_1b_R, V_2_R, discussed in sections below) [103] and its short systemic half-life [104]. More systematic studies and OTR drug lead development is, in our opinion, necessary before moving towards clinical translation.

### In vitro studies

OT has antiproliferative effects in several human breast cancer cell lines, including MDA-MB-231 [40, 94], MCF-7 and T47D [40]. However, experimental conditions varied and some studies yielded contradictory data (Table 2). Multiple factors can affect OTR expression. For example, OTR expression in both ER^+^ (MCF-7) and ER^-^ (Hs578T) breast cancer cell lines can be increased by E2 treatment and decreased by progesterone treatment [87]. In the breast cancer Hs578T cell line, serum deprivation results in loss of OTR and serum restoration and addition of 1 μM dexamethasone increases OTR mRNA levels by 9-fold [90]. The variability in OTR expression induced by hormones, serum and potentially other environmental changes may partially explain the contradictory results [87, 90]. There are hormones (including E2 and OT) and growth factors present in serum, therefore, using charcoal-stripped FBS (CS-FBS) in the proliferation assay, which can reduce effects of the hormones and growth factors in FBS [105], could be a critical factor to consider in the in vitro study of the OT/OTR system.

**Table 2:** Effects of OT analogues on breast cancer cell proliferation in vitro.

Cell lines	Ligands	Conditions	Effects	Reference
HMEC, B-TEC	OT, 10^−9^–10^−6^ M	10% FCS or serum free, medium changed every 48 h	Stimulation (proliferation & migration)	[91]
MCF-7	OT, 10^−11^–10^−9^ M	2.5% FCS	Stimulation	[59]
	VP, 10^−8^ M		Inhibition	
	VP, 10^−11^–10^−9^ M		Stimulation	
MCF-7	OT, 10^−7^ M	2% FCS in medium with E2 or CS-FCS in phenol red-free medium	Inhibition	[92]
MCF-7, SK-BR-3	VP, 10^−8^ M	5% CS-FCS	Stimulation	[93]^a^
	Desmopressin, 10^−8^ M		Inhibition	
MDA-MB-231	OT, 10^−7^ M	10% FCS	Inhibition	[94]
MDA-MB-231, MCF-7	OT, 10^−7^ M	10% FCS for 5 days	Proliferation inhibition, differentiation stimulation	[40]
MDA-MB-231	OT, 10^−9^ M, 10^−8^ M, 10^−7^ M AT, 10^−8^ M, 5 × 10^−8^ M	5% FCS, medium changed every 24 h	Inhibition	
MCF-7, T47D	OT, 10^−7^ M, 10^−8^ M	5% FCS, medium changed every 24 h	No effects	
		10% FCS, E2, TAM, medium changed every 24 h	Inhibition	
MDA-MB-231, MDA-MB-361, MDA-MB-468, MCF-7	OT, 10^−9^ M, 10^−7^ M	5% FCS	No effects	[88]
Hs578T	OT, unspecified concentration	Not available	No significant short-term effects	[90]
MCF7, TS/A (mouse)	LVT, 10^−8^ M, 10^−7^ M 10^−6^ M DOTA-LVT, 10^−8^ M, 10^−7^ M 10^−6^ M	10% FCS	Inhibition No effects	[100]^b^
MDA-MB-231 TS/A (mouse)	OT, 10^−8^ M, 10^−7^ M	10% FCS	Inhibition	[68]
TS/A (mouse) D-R3230AC (rat)	OT, AT, 10^−8^ M	10% FCS	Inhibition	[96]
CMT-U27 (canine)	OT, Desmopressin, 10^−6^ M	10% FBS	Inhibition	[153]

*HMEC* human dermal microvascular endothelial cells, *B-TEC* tumour-associated endothelial cells purified from human breast carcinomas, *FCS (FBS)* fetal calf (bovine) serum, *CS-FCS* charcoal-stripped FCS, *E*2 17β-estrodial, *TAM* tamoxifen, *AT* atosiban, *OT* oxytocin, *VP* vasopressin, only two amino acids different from OT at position 3 and 8, functions via vasopressin receptors V_1a_R, V_1b_R, and V_2_R, also can activate OTR; *Desmopressin* 1-(3-mercaptopropionic acid)−8-D-arginine-vasopressin (V_1b_R/V_2_R agonist), an analogue of VP with longer half-life and improved selectivity for V_2_R; LVT, Lys^8^-vasotocin, *DOTA-LVT* 1,4,7,10-tetraazacyclododecane-N,N’,N”,N”’-tetraacetic acid (DOTA) Lys^8^-vasotocin.

^a^Study focused on VP and its receptors.

^b^Study developed a radio-labelled ligand targeting OTR-expressing tumours, proliferation effects of the two ligands were also assessed.

It is also worth noting that most of these studies used human breast cancer cell lines that are not derived from primary, untreated breast tumours, but from advanced-stage, often pre-treated, tumours, metastases, and pleural effusions. For example, SK-BR-3, MDA-MB-231, MCF-7, T47D and MDA-MB-468 are all derived from metastatic lesions’ pleural effusions, and MDA-MB-361 are derived from a metastatic breast cancer site in the brain. Thus the results obtained are likely to be more indicative of rapidly progressive types of breast carcinoma and late-stage disease, rather than lower grade and earlier stages of breast cancers [106]. The outcomes of these in vitro studies highlight the need for a more systematic approach to study OTR as a target with highly reproducible experimental conditions and appropriate controls, including thorough monitoring and characterisation of the breast cancer cell lines used, such as their OTR expression levels and signalling pathways to provide a deeper and more controlled understanding of the mechanisms underlying these conflicting effects.

## Basic molecular biology of the OTR and its signalling pathways in breast cancer

OTR comprises three extracellular loops (EL1-EL3), seven transmembrane α-helices (7TM) and three intracellular loops (ICL1-ICL3) [107], with the N-terminus at the extracellular and the C-terminus the intracellular side. The intracellular domains and loops of OTR couple to G protein α, β and γ subunits, which are at the top of the signalling cascade. Ligands bind to OTR in an extracellularly accessible membrane binding pocket, which induces a conformational change leading to the recruitment of G proteins.

Upon receptor activation (e.g., via OT), OTR can signal via multiple G protein-dependent and G protein-independent pathways that activate signalling cascades including a variety of second messenger systems, as well as members of the Ras and Rho families of small GTP-binding proteins. These pathways have multiple effects and understanding of the various signalling cascades is important, as it might provide clues on its role in breast cancer as well as potential targets for pharmacotherapy. The impact of OTR signalling via G protein-dependent/-independent and downstream effector proteins is thus discussed in the following paragraphs, particularly in terms of molecular mechanisms influencing breast cancer cell proliferation and migration.

### G protein-dependent signalling of OTR

**Fig. 2:**
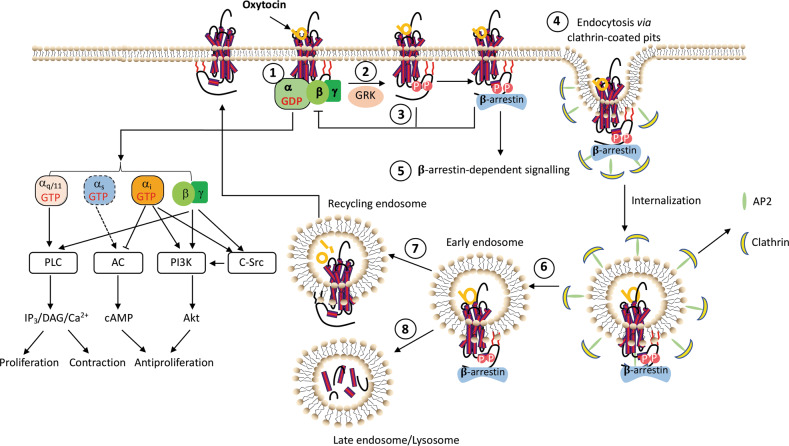
Schematic pathway diagram of G protein-dependent and β-arrestin-dependent signalling via OTR. Upon agonist binding: (1) OTR signals via G proteins—the classic signalling route for Gα_q/11_ is activation of phospholipase C (PLC) thereby triggering phosphoinositide hydrolysis, calcium mobilisation and protein kinase A (PKA) activation. Gα_s_ activates adenylate cyclase (AC), which in turn increases cAMP production, leading to the activation of cAMP-dependent PKA. The OTR/Gα_s_ pathway (dashed lines) is not fully confirmed yet and requires further study. The classical signalling pathway for Gα_i_ is inhibition of AC which leads to a decreased cAMP production and decreased PKA activity. (2) In addition, OTR can be phosphorylated at the C-terminus or at intracellular loops by GPCR kinases (GRK). (3) Once phosphorylated, OTR loses its affinity for the G proteins and gains affinity for β-arrestins. β-arrestins bound to OTR prevent further coupling of G proteins, a process known as desensitisation. (4) β-arrestins, through their interaction with clathrin and adaptor protein 2 (AP-2), target phosphorylated OTRs for endocytosis via clathrin-coated pits by scaffolding proteins of the internalisation machinery. (5) OTR can also activate β-arrestin-dependent signalling pathways. (6) Internalised OTRs move into early endosomes, of which some are sorted into recycling endosomes, where the ligand is metabolised and the receptors are dephosphorylated and recycled back to the cell surface (7), while others are sorted to late endosomes/lysosomes for degradation (8).

OT and other OTR agonists promote the replacement of guanosine-5′-diphosphate (GDP) with guanosine-5′-triphosphate (GTP) to interact with the Gα subunit, subsequently causing the dissociation of Gα from Gβγ subunits and ultimately affecting different downstream effectors or signalling events. OTR can engage in vitro with Gα_q/11_, Gα_i/o_ and Gα_s_, which further signal through their second messengers IP_3_ (inositol 1,4,5-trisphosphate), DAG (diacylglycerol), Ca^2+^ or cAMP (cyclic adenosine monophosphate) to trigger a multitude of intracellular signalling events (Fig. 2) [108–111].While OTR Gα_q/11_ and Gα_i/o_ recruitment and signaling is well established, OTR/Gα_s_ signaling remains questionable, with only low [84] or no significant Gα_s_ recruitment [99] observed.

Activation of the Gα_q/11_ pathway might be a critical factor for proliferative effects of OT since it induces an increase of IP_3_ or intracellular calcium ([Ca^2+^]_i_), which further promotes protein tyrosine phosphorylation—an early event of proliferative signalling triggered also by growth factors [13, 15]. For example, the increase in [Ca^2+^]_i_ plays a role in the stimulation of cell proliferation in small-cell lung cancer [12], trophoblast and choriocarcinoma [13], and Kaposi’s sarcoma [15]. However, the effects of these OT-induced intracellular changes vary across different breast cancer cell lines, for example, leading to an increase of total inositol phosphates in MCF-7 cells resulting in proliferation [59], no change of [Ca^2+^]_i_ in MDA-MB-231 cells with proliferation inhibition associated with cAMP signalling [94], no change of [Ca^2+^]_i_ in T47D cells [112] with antiproliferative effects [40], and a time- and concentration-dependent increase in [Ca^2+^]_i_ in Hs578T cells with no significant effects on proliferation [90]. Mitogenic effects of OT on MCF-7 [59], HEMC and B-TEC cells [91] were also associated with Gα_q/11_ pathway activation resulting in IP_3_ or [Ca^2+^]_i_ increase. Gβγ subunits released by OTR/Gα_q/11_ activation can lead to ERK1/2 activation in myometrial cells via a PLC-independent pathway, involving EGFR activation and calcium requirement [113]. This might help to explain some of the proliferation effects of OT in breast cancer cell lines with changes in IP_3_ or [Ca^2+^]_i_ signalling.

Low amounts of Gα_s_ complexation with OTR was observed in rat myometrium [84], but such a Gα_s_ interaction was not confirmed in a bioluminescence resonance energy transfer (BRET) study looking at G protein recruitment at the human OTR [99]. In the triple-negative MDA-MB-231 cell line, OT displayed antiproliferative effects associated with an increase in intracellular cAMP levels and activation of PKA, with no involvement of the Ca^2+^-phosphoinositide system, which might be linked to the Gα_s_ pathway [94]. In Hs578T cells, OT promoted the synthesis of prostaglandins [90], which may indirectly involve the activation of Gα_s_-coupled prostaglandin receptors [114]. At this stage, the OTR/Gα_s_ pathway remains questionable and requires further investigation.

OTR coupling to Gα_i_ is especially crucial for cell growth inhibition since pertussis toxin (PTX) treatment, which inhibits Gα_i_ protein-receptor coupling, entirely abolished the inhibitory effects mediated by GFP-tagged OTR stimulation. Meanwhile, inhibition of PLC with its inhibitor U73122 also abolished the inhibition of cell growth by OTR coupling to Gα_i_, which is possibly a result of PLC activation by the βγ complexes released by Gα_i_ [115]. The Gα_i_ pathway appears to also mediate breast cancer growth inhibitory effects of OT, with the involvement of the PI3K/Akt pathway [56, 97]. In MCF-7 cells, which express OTR and feature a PI3K activating mutation, a 30 min incubation with OT (125–1,000 nM) reduced the abundance of PI3K signalling components, including a reduction of PI3K p85, PI3K p110, and pAktS^473^, and Akt phosphorylation downstream of OTR requires Gα_i_ and a structural change in the receptor [116]. Gα_i_ and βγ complexes are also probably involved in mediating c-Src and PI3K activation [115] which play crucial roles in breast cancer.

More systematic studies, including a range of well-defined and breast cancer subtype-related cell lines, are required to determine the functional role of modulating OTR and its downstream signalling pathways during disease development and progression.

### G protein-independent signalling of OTR

OTR also mediates G protein-independent signalling, which is less studied than G protein-dependent signalling. OTR stimulation and subsequent phosphorylation by GPCR kinases (GRKs) relay the primary steps in the induction of G protein-independent signalling by inducing the recruitment of β-arrestins. While β-arrestins desensitise G protein-mediated GPCR signalling, they also elicit G protein-independent signalling cascades, promoting cellular responses such as cell migration and proliferation [117]. For OTR, β-arrestins act as essential multifunctional adaptors in receptor desensitisation and trafficking, and some OT-induced OTR signalling depends on β-arrestins, such as the MAPK pathway [118].

OTR can bind both β-arrestin-1 and -2, forming a stable OTR/β-arrestin complex, which internalises into endosomes. Some early endosomes are sorted into recycling endosomes, where the ligand is metabolised and OTR dephosphorylated and recycled back to the plasma membrane, while others are sorted to late endosomes/lysosomes for degradation (Fig. 2) [119, 120]. OTR with OT bound to it has a higher potency for β-arrestin-2 than for β-arrestin-1 (BRET measurements of OTR-mediated β-arrestin-1 and β-arrestin-2 recruitment induced by OT stimulation: EC_50_ of 41.15 ± 1.85 nM for β-arrestin-2 and 229 ± 23.15 nM for β-arrestin-1, 2 min for β-arrestin-2 and 5 min for β-arrestin-1 to reach BRET signal maximum plateau level) [99]. While β-arrestin binding can physically obstruct G protein coupling, there exist also additional mechanisms by which β-arrestins block of G protein signalling [121]. OTR internalisation and desensitisation are induced upon agonist exposure through the classical clathrin-coated pits via β-arrestins (Fig. 2) [120]. For example, OT-induced internalisation of OTR occurs rapidly in a time- and OT dose-dependent manner (e.g., 100 nM OT, half-time of surface receptor loss is 2.3 min, reaching ~75% cell surface receptor loss after 1 h) [120]. OTR internalisation and desensitisation has been studied and exploited for breast tumour visualisation and targeted treatment strategy in OTR-positive cancers [100, 101]. For example, radio-labelled OT analogues are efficiently taken up by OTR-expressing TS/A mammary carcinoma cells both in vitro and in vivo [100, 101], and a macromolecular conjugate of paclitaxel bearing OT as a targeting moiety was successfully delivered into OTR-positive breast cancer MCF-7 cells [122].

Receptor dimerization, compartmentalisation and trafficking, as well as receptor-transducer-effector stoichiometry and ligand residence and exposure times can each affect OTR coupling. Extrinsic factors, such as cell type or assay conditions, can also influence receptor signalling. Considering the complexity and heterogenicity of breast cancer and OTR signalling, it is critical to take the molecular characteristics of different breast cancer subtypes and cell types into consideration when studying OTR-mediated pathways in breast cancer. In addition, it is important to acquire a more comprehensive understanding of the physiological and pathological consequences of both G protein-dependent and β-arrestin-dependent signalling (including β-arrestin-1 and -2 pathways), particularly when it comes to targeting OTR in breast cancer.

### Biased signalling

OTR can promiscuously engage with multiple G proteins and initiate various signalling pathways in different cell systems. OT can activate Gα_q_, all members of the Gα_i/o_ families including Gα_i1_, Gα_i2_, Gα_i3_, Gα_oA_, and Gα_oB_ (EC_50_ of OT for activation of different OTR-G protein complexes in HEK293 cells by BRET: Gα_q_, 2.2 nM; Gα_i3_, 11.5 nM; Gα_oA_, 29.8 nM; Gα_i2_, 32.3 nM; Gα_i1_, 62.6 nM and Gα_oB_, 91.8 nM) [99], and maybe Gα_s_ [84]. It is possible to identify ligands to direct signalling towards a selected G protein-dependent or G protein-independent downstream signalling pathway, a concept called ‘biased signalling’ [123, 124]. Biased signalling can be encoded through three general mechanisms including biased ligand, biased receptor, or biased system [124]. OTR can elicit specific signalling pathway transduction via biased ligands and via its localisation within caveolae.

The well-characterised OT analogues AT and DNalOVT [125] are biased ligands that block the Gα_q/11_ pathway while activating the Gα_i3_ (AT) or Gα_i1_ (DNalOVT) pathways [99]; another example is carbetocin, a functional selective Gα_q/11_ agonist promoting OTR internalisation via a β-arrestin-independent manner [126]. Such biased ligands are valuable probes to dissect the complex OTR-dependent signalling pathways, particularly when it comes to OTR’s involvement in (breast) cancer. For example, AT, which favours Gα_i/o_ over Gα_q/11_ coupling [98], significantly inhibits rat and mouse breast tumour growth both in vitro and in vivo [68, 96]. Indeed, OTR coupling to Gα_i_ seems to be especially important in mediating the inhibition of cell growth [115], and independent activation of either Gα_i1_ (by DNalOVT) or Gα_i3_ (by AT) can lead to growth inhibition in cells expressing a functional Gα_i_-coupled OTR (HEK293 cells stably expressing the OTR-enhanced green fluorescent protein and DU145 human prostate cancer cells expressing endogenous OTR) [99]. These selected Gα_i_ pathway activation studies with AT and DNaIOVT are the first experimental evidence that activation of the OTR-Gα_i_ pathway could be responsible for the observed tumour growth inhibition effects [98].

In addition to G protein and β-arrestin, localisation of OTR in caveolae can also induce biased signalling. Caveolae are small pits of 50–60 nm in diameter on the plasma membrane, with peripheral membrane proteins called cavins coating the caveolar surface and caveolins embedded in the interior membrane layer as the main structural components [127]. OTR does not undergo internalisation through the clathrin-mediated route when it is located in the caveolae. It activates the MAP kinase in a Ras-dependent pathway leading to transient activation (<30 min) and translocation of EGFR/ERK1/2 and stimulates cell proliferation, and this may be due to the increased affinity of OTR coupled to Gα_q/11_, because PTX pre-treatment did not block the mitogenic effect. When OTR is outside the caveolin-enriched domains, its activation leads to sustained activation (>3 h) of EGFR/ERK1/2 through a Gα_i_-, PLC-, c-Src- and PI3K-independent pathway, altering its regulatory effects on proliferation towards antiproliferative, by engaging the cell cycle inhibitor p21WAF1/CIP1 [114, 115].

Biased signalling could be a reason for observing different proliferation results (proliferative, antiproliferative and no effects) in response to OTR activation. Indeed, the findings discussed above point towards targeting specific OTR pathways for clinical application, indicating that the Gα_i_ pathway in particular might play an important role in disease progression. Investigation of the specific OTR pathways with well-characterised biased ligands is thus encouraged to advance our understanding of OTR in breast cancer development and progression.

## Links between the OTR and the oestrogen receptor in breast cancer

OTR also interacts with other proteins, some of which are highly relevant and dysregulated in breast cancer, such as the oestrogen receptors (ERs), which are present in approximately 75% of all breast cancer cases. ERs include two subtypes, ERα and ERβ, encoded by ESR1 and ESR2, respectively, which can function at the membrane or within the nucleus [128]. ERα is the most studied subtype due to its clinical significance, while the clinical relevance of ERβ remains obscure [129]. The abbreviation ER thus refers mainly to ERα in the text below as well as in the literature. ER responds to oestrogen, which exists endogenously as three major forms in females, namely estrone (E1), estradiol (E2), and estriol (E3), another type of oestrogen, estetrol (E4), is specific to pregnancy. In breast cancer epithelial cells, the mitogenic action of E2 is mediated via ERα and ERβ [130].

OTR expression is modulated by E2. Increased OTR mRNA expression is observed not only in normal mammary myoepithelium but also in human breast cancer cell lines MCF-7 (ER^+^) and Hs578T (ER^−^) that were exposed to E2 (10^–7^ M or 10^–6^ M, 24 h) [87]. In MCF-7 and T47D (ER^+^) cell lines, 30 min of 10 nM E2 treatment yielded a 100-fold increase in the OTR mRNA expression [37]. However, the correlation between OTR expression and ER^+^ breast tumours is not consistent among studies [58, 68, 88]. The increased OTR levels with E2 dominance indicate that there is a potential for these tissues to be sensitive to OTR-mediated effects, including inhibition of tumour growth [131]. For example, OT inhibits E2-induced growth of MCF-7 and T47D cells, and increases the antiproliferative effects of tamoxifen in these ER^+^ cells [40]. Conversely, OT can also influence tumour growth indirectly, by affecting ER expression or function. Accordingly, OT treatment in MCF-7 cells downregulates ER and therefore the oestrogen-dependent mitogenic response [92]. Multi-drug-resistant breast cancer cell lines also feature OTR overexpression [132]. Therefore patients with ER^+^ breast tumours, which have intrinsic or acquired resistance for endocrine manipulation, are likely to benefit from an OTR-targeting therapeutic approach.

The correlation between OTR and ER and its significance remains to be clarified further with larger sample sizes; however, an OTR-targeting therapy might display synergistic effects with an ER-targeting therapy and could contribute to the battle against drug resistance of ER^+^ breast cancer.

## Bottlenecks and strategies of targeting the OTR for breast cancer management

As a GPCR, OTR is a highly druggable target, and targeting OTR in breast cancer could lead to improved treatment and diagnostic options of this heterogeneous disease. Nonetheless, there is still no OT analogues undergoing clinical trials for cancer treatments. Broader and more systematic studies are required to define the therapeutic potential of OTR in breast cancer. In the following section, we discuss current bottlenecks as well as potential drug development strategies.

### Bottlenecks for targeting OTR in breast cancer

**Fig. 3:**
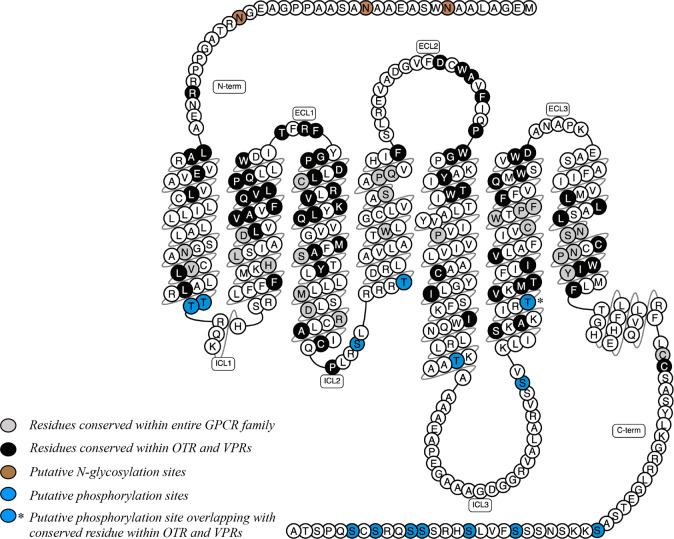
The structure and sequence of human OTR, with amino acids presented as single-letter codes. Residues conserved within OTR and VPRs are coloured in black, residues conserved within the entire family of GPCRs are coloured in grey, putative N-glycosylation sites are coloured in brown, putative phosphorylation sites are coloured in blue, and putative phosphorylation sites overlapping with conserved residue within OTR and VPRs are coloured in blue and marked with an asterisk. The receptor scheme was adapted from GPCRdb website [133], the conserved residues were adapted from [4], and the putative phosphorylation sites were adapted from [111].

Vasopressin (VP) with its three receptors (VPRs), V_1a_R, V_1b_R, and V_2_R, are closely related to OT/OTR, constituting one of the most complex and important neuroendocrine systems in humans. Human VP is highly homologous to OT, with only two amino acids different at positions 3 and 8 (VP, CY**F**QNCP**R**G). Members of the OT/VP-receptor family are highly conserved sharing a high degree of sequence similarity, about 100 conserved amino acids among the 370–420 amino acids, especially in the extracellular loops and the transmembrane helices (Fig. 3) [4, 8, 133]. The homology of the binding sites of the OTR and VPRs is ~80%, thus leading to significant cross-talk [103, 134, 135]. OT can not only activate OTR, but also the VPRs, while VP can also activate OTR. Hence, if OT and OT analogues inhibit proliferation, it cannot be simply concluded that this occurs exclusively via OTR—since VPRs might also be present and play a role. OTR-selective ligands are thus critical to define the role of OTR in breast cancer [99, 103, 125, 136]. Receptor-selective ligands are also important for drug development, since unspecific off-target effects can lead to undesired side effects (e.g., V_1a_R regulates cardiovascular functions, V_2_R regulates kidney functions) [137, 138].

Another barrier for studying the OT/OTR system in breast cancer is the lack of OTR *protein* expression data in human tissues. Most OTR expression data in breast cancer tissues are on *gene* expression levels determined by PCR and in situ hybridizations [37, 40, 68, 139], and there are several more gene expression data sets being generated by a variety of high-throughput hybridization array- and sequencing-based techniques (e.g., RNA-seq and ChIP-seq), accessible through databases such as Gene Expression Omnibus (GEO) [140]. However, gene expression levels are not always indicative of protein abundance. The high homology of the extracellular loops of the receptors is a major problem for OTR/VPR antibody selectivity, which, to date, is still unsatisfying for OTR protein detection. All of the OT/VP receptors have putative glycosylation sites at the N-terminus (Fig. 3) [141], which is another hurdle for OTR protein detection [142], since it induces a variation of OTR’s molecular mass, rendering detection by western blotting ambiguous and often inconclusive [142]. This results in limited and reliable OTR protein expression data to date [78].

Even though biased signalling might explain some of the contradictory data on OT analogues in breast cancer growth, it is not always clear which specific pathway mediates a beneficial or adverse therapeutic response [123]. Identification of the relevant signalling pathways for breast cancer is however critical as this will form the basis for a more directed, if not a rational, ligand design towards novel treatment options.

Breast cancer, like many other cancers, is not just a single disease but a group of biologically and molecularly heterogeneous disease subtypes [143], each characterised by distinct morphology, biomarkers, behaviour and clinical implications. It is thus important to further explore the therapeutic potential of the OT/OTR system in breast cancer and expand these studies across a broader range of breast cancer subtypes. It also remains unclear how an acute or single dose of OT analogues compares versus a long-term continuous administration affects in terms of breast cancer growth. Therefore, elucidating the distribution of OTR under physiological and specific pathological conditions in human breast and their corresponding precise molecular signalling pathways should be a continuing research focus.

### Strategies for targeting OTR in breast cancer

The demonstrated druggability of OTR and its involvement in breast cancer provides a driving force for drug development in this field. Many OT-like peptides have been developed with improved selectivity and more drug-like properties [125, 144]. Bioactive OT/VP-like peptides from natural sources form promising starting points for the identification of pharmaceutical lead compounds, a strategy that our laboratories have pursued now for many years [7]. It takes advantage of the evolutionary conservation of this signalling system across the animal kingdom and provides high hit-rates for the discovery of bioactive OT ligands with often unique and therapeutically relevant pharmacology [8, 136, 145–147]. These ligands play an important role in dissecting these cancer-related signalling pathways and, combined with state-of-the-art medicinal chemistry, also form exquisite starting points for drug development programs [103, 148–151].

Biased signalling is gaining prominence, which raises the possibility of directly screening for OTR-specific G protein-dependent or independent pathway modulators. These ligands will play an important role in the dissection of OTR signalling pathways and function as well as in advancing our understanding of the pathway-specific role in breast cancer progression. One of the most commonly used techniques to study GPCR pathways signal transduction is BRET, which is currently the preferred technique to identify and characterise biased ligands for the OTR signalling system [99].

## Conclusions and future directions

Accumulating evidence underpins the involvement of the OT/OTR signalling system in breast cancer development. This warrants further investigation, ideally in a systematic and well-controlled way using state-of-the-art techniques. The use of receptor-subtype selective, if not biased, ligands is critical to study the preventive and tumour reducing capabilities of the OT/OTR signalling system in breast cancer. A thorough understanding of OTR’s signalling pathways in controlling breast cancer formation and progression is mandatory to understand the pathological process and to design an OTR-based therapeutic approach. From a drug development point of view, OTR represents a promising therapeutic target for breast cancer diagnosis and treatment that is worth pursuing.
